# Cost-utility and biological underpinnings of Mindfulness-Based Stress Reduction (MBSR) versus a psychoeducational programme (FibroQoL) for fibromyalgia: a 12-month randomised controlled trial (EUDAIMON study)

**DOI:** 10.1186/s12906-016-1068-2

**Published:** 2016-02-27

**Authors:** Albert Feliu-Soler, Xavier Borràs, María T. Peñarrubia-María, Antoni Rozadilla-Sacanell, Francesco D’Amico, Rona Moss-Morris, Matthew A. Howard, Nicolás Fayed, Carles Soriano-Mas, Marta Puebla-Guedea, Antoni Serrano-Blanco, Adrián Pérez-Aranda, Raffaele Tuccillo, Juan V. Luciano

**Affiliations:** Teaching, Research & Innovation Unit, Parc Sanitari Sant Joan de Déu, C/Dr. Antoni Pujadas 42, 08830, Sant Boi de Llobregat, Barcelona, Spain; Centre for Biomedical Research in Mental Health, CIBERSAM, Madrid, Spain; Stress and Health Research Group, Faculty of Psychology, Universitat Autònoma de Barcelona, Bellaterra, Barcelona, Spain; Primary Health Centre Bartomeu Fabrés Anglada, DAP Delta Llobregat, Unitat Docent Costa de Ponent, Institut Català de la Salut, Gavà, Spain; Primary Care Prevention and Health Promotion Research Network (RedIAPP), Madrid, Spain; Rheumatology Service, Parc Sanitari Sant Joan de Déu, Sant Boi de Llobregat, Spain; Personal Social Services Research Unit, London School of Economics and Political Science, London, UK; Health Psychology Section, Department of Psychology, Institute of Psychiatry, Psychology, & Neuroscience, King’s College London, London, UK; Department of Neuroimaging, Institute of Psychiatry, Psychology & Neuroscience, King’s College London, London, UK; Magnetic Resonance Unit, Department of Radiology, Hospital Quironsalud Zaragoza, Zaragoza, Spain; Department of Psychiatry, Bellvitge University Hospital, Bellvitge Biomedical Research Institute (IDIBELL), Barcelona, Spain; Department of Psychobiology and Methodology of Health Sciences, Universitat Autònoma de Barcelona, Barcelona, Spain; Aragon Institute of Health Sciences (I+CS), Zaragoza, Spain; Hospital de Sant Pau, Barcelona, Spain

**Keywords:** Fibromyalgia, Mindfulness-Based Stress Reduction, Psychoeducation, Neuroimaging, Cytokines, Cost-utility

## Abstract

**Background:**

The EUDAIMON study focuses on fibromyalgia syndrome (FMS), a prevalent chronic condition characterized by pain, fatigue, cognitive problems and distress. According to recent reviews and meta-analyses, Mindfulness-Based Stress Reduction (MBSR) is a promising therapeutic approach for patients with FMS. The measurement of biomarkers as part of the analysis of MBSR effects would help to identify the neurobiological underpinnings of MBSR and increase our knowledge of FMS pathophysiology. The main objectives of this 12-month RCT are: firstly, to examine the effectiveness and cost-utility for FMS patients of MBSR as an add-on to treatment as usual (TAU) versus TAU + the psychoeducational programme FibroQoL, and versus TAU only; secondly, to examine pre-post differences in brain structure and function, as well as levels of specific inflammatory markers in the three study arms and; thirdly, to analyse the role of some psychological variables as mediators of 12-month clinical outcomes.

**Methods:**

Effectiveness, cost-utility, and neurobiological analyses performed alongside a 12-month RCT. The participants will be 180 adult patients with FMS recruited at the Sant Joan de Déu hospital (St. Boi de Llobregat, Spain), randomly allocated to one of the three study arms: TAU + MBSR vs. TAU + FibroQol vs. TAU. A comprehensive assessment to collect functional, quality of life, distress, costs, and psychological variables will be conducted pre-, post-intervention, and at 12-month post-intervention. Fifty per cent of study participants will be evaluated at pre- and post-treatment using Voxel-Based Morphometry, Diffusion Tensor Imaging, pseudo-continuous Arterial Spin Labeling, and resting state fMRI. A cytokine multiplex kit of high-sensitivity will be applied (cytokines IL-6, IL-8, IL-10 + high-sensitivity CRP test).

**Discussion:**

The findings obtained from this RCT will indicate whether MBSR is potentially cost-effective for FMS and contribute to knowledge of any brain and inflammatory changes associated with MBSR in FMS patients. Specifically, we will determine whether there are morphometric and functional changes associated with participation in MBSR in brain regions related to meta-awareness, body awareness, memory consolidation-reconsolidation, emotion regulation and in networks postulated to underpin the sensory-discriminative, cognitive-evaluative and affective-motivational aspects of the pain experience.

**Trial registration:**

NCT02561416. Registered 23 September 2015.

## Background

Fibromyalgia (FMS) is a debilitating syndrome usually diagnosed in women between the ages of 20 and 50 years [1]. It is characterised by multifocal pain, fatigue, disturbed sleep, cognitive problems, and high levels of distress [2]. A survey performed in five European countries indicated that the estimated overall prevalence of FMS is 2.9 % in the general population [3]. Patients with FMS typically present comorbidities with psychiatric disorders such as anxiety (13–63.8 %) and depressive disorders (20–80 %) [4]. In addition, FMS is associated with high direct (medical visits, multiple prescriptions of medications, etc.) and indirect (absenteeism, work loss, etc.) costs in industrialised countries [5]. Among chronic pain conditions, FMS causes the highest rates of unemployment, the highest rate of incapacity benefits claim rate, and the greatest number of days absent from work [6]. FMS represents a great challenge for health professionals because of the lack of optimal treatment options. The effectiveness of pharmacological interventions is generally limited and more ubiquitous effects have been found for non-pharmacological treatments [7].

The Central Nervous System (CNS) seems to play a crucial role in the pathogenesis of FMS [1, 8]. In this regard, structural and functional brain abnormalities in several areas related to pain and stress response regulation (e.g., anterior cingulate cortex, insula, parahippocampal gyrus, prefrontal cortex, somatosensorial cortex) have been observed in patients with FMS. Specifically, significant grey matter reductions in the prefrontal cortex, anterior cingulate cortex and insular cortex have been reported [9]. Such alterations may contribute to the impaired control of pain and abnormal processing of painful stimuli [8]. Alterations in functional connectivity within the brain’s pain inhibitory network; the default-mode network (DMN) and the executive attention network (EAN), as well as greater intrinsic connectivity between the insula and the DMN and EAN were also reported [10, 11]. Furthermore, higher spontaneous pain at the time of the scan was found to be correlated with greater intrinsic connectivity between the insula and both the DMN and the EAN [11]. Moreover, reductions in insula-DMN connectivity were associated with pain reduction in FMS [12], suggesting a key role in the etiology of the disease. Reduced resting connectivity within the somatosensory system and increased connectivity between the DMN and somatosensory processing regions such as S2 have been recently reported [13], which may suggest that a general weakening of sensory integration may be also underlie clinical pain in patients with FMS.

In addition to the aforementioned abnormalities in the CNS, imbalances in inflammatory markers have also been also observed in FMS. Specifically, higher levels of pro-inflammatory cytokines (IL1, IL6, and IL8) and lower levels of anti-inflammatory cytokines (IL4 and IL10) have been reported [14–16]. This cytokine imbalance may produce a chronic inflammatory state in the CNS and the peripheral nervous system, facilitating the sensitisation of peripheral nerves to nociceptive stimuli, increasing hypothalamic-pituitary-adrenal axis activity and the synthesis of prostaglandin and substance P, which lower the pain threshold [14]. Furthermore, cytokines have further effects on the CNS by altering synthesis, reuptake, and release of neurotransmitters involved in the perception, and affective, cognitive and motivational regulation of pain, contributing to structural and functional brain changes and to the perpetuation of pain [17]. Additionally, pro-inflammatory cytokines augment tryptophan metabolism, which may boost affective symptoms in FMS [18–20] and activate glial cells that release a combination of substances into the cerebral spinal fluid that are associated with pain amplification (P substance, glutamate, nerve growth factor, and brain-derived neurotrophic factor) [21].

Contemplatives have pointed out that the practice of meditation reduces the experience of pain by controlling expectations, the nature and orientation of attention towards the experience, and its related emotional response. Mindfulness-based interventions (MBIs) were conceived in the late 1970s from the effort to integrate Buddhist meditation into western psychological practice. In the last decade, there has been growing interest in the effectiveness of MBIs for a range of physical and mental conditions [22], including chronic pain. Veehof et al. [23], in a meta-analysis in chronic pain patients, showed that MBIs have significant effects on pain intensity, depression, anxiety, physical wellbeing, and quality of life. Given that there are no curative treatments available for patients with FMS [1], the putative use of MBIs as coadjuvants of usual care is highly promising.

Bawa and colleagues [24] recently found limited evidence for the effectiveness of MBIs in chronic pain. Their meta-analysis revealed that MBIs have a positive impact on perceived pain control although there was no evidence of a benefit in clinical outcomes. Separate subgroup analysis found evidence of improved physical functioning and quality of life for MBIs versus inactive control conditions. When compared to active control conditions, the effect of MBIs was equivalent to the active comparator. However, results were inconclusive given that the included studies generally involved small samples. In line with these findings, a systematic review of 10 studies [25] indicated that although MBIs provide some significant physical and psychological benefit for patients with FMS, the divergence in the outcomes measured, sample sizes, and data presented failed to establish definitive conclusions about the effectiveness of MBIs. The specific effectiveness of Mindfulness-Based Stress Reduction (MBSR) for FMS patients was evaluated by Lauche and colleagues [26], who found that MBSR improves quality of life and reduces pain in the short term. Nevertheless, further high-quality RCTs are still necessary because of the considerable methodological limitations of the meta-analysed studies (e.g., absence of randomization, high attrition rates, or small sample sizes).

There is a great amount of evidence of structural changes in experienced meditators compared to individuals without meditative experience [27, 28]. These changes would be indicative of increases in meta-awareness (frontopolar cortex), exteroceptive and interoceptive body awareness (sensory cortices and insula), memory consolidation and reconsolidation (hippocampus), self and emotion regulation (anterior, mid cingulate and orbitofrontal cortices), and intra- and interhemispheric communication (superior longitudinal fasciculus, corpus callosum) associated with meditation. Structural changes in specific brain areas (e.g., hippocampus, posterior cingulate cortex, the temporo-parietal junction, and cerebellum) have been reported after a MBSR intervention [29].

Significant differences in brain function have been observed between meditators and non-meditators. In this regard, Brewer et al. [30] found reduced activation of two main DMN nodes (posterior cingulate cortex and medial prefrontal cortex) and activations of the medial prefrontal cortex, insula, and temporal lobes during meditation in individuals with meditative experience, showing a differential pattern of functional connectivity both during resting and when practicing mindfulness. Similarly, Jang et al. [31] reported greater functional connectivity (at rest) in some areas of the DMN (i.e., the medial prefrontal cortex) in individuals with meditative experience, suggesting that meditation practice would be associated with functional changes in DMN areas even when not practicing meditation. Furthermore, functional brain changes (e.g., increases in left frontal activation, higher activity in insula, secondary somatosensorial cortex, anterior cingulate cortex, lower activity in right amygdala and in several DMN regions) have been observed after standard MBSR [32]. All the brain changes following mindfulness training are associated with increased learning and memory processes, emotion regulation, self-referential processing, and perspective taking, an augmented experiential, present-focused mode of self-reference, higher interoceptive awareness, more accurate processing of exteroceptive sensory events, higher attentional control and reduced conceptual processing [29, 32, 33]. Interestingly, mindfulness training seems to impact on pain experience as significantly lower pain unpleasantness and intensity ratings (compared to resting state) were found after a brief mindfulness training session when meditating in the presence of painful stimulation [34]. In this study, reductions in pain intensity ratings were found to be associated with increased activity in areas involved in the cognitive and affective regulation of nociceptive processing (anterior cingulate cortex and insula) and reductions in pain unpleasantness ratings were associated with increased activity in brain areas (orbitofrontal cortex) involved in reframing the contextual evaluation of sensory events and diminished thalamic activity, which may reflect a change in the interaction between afferent input and executive-order brain areas.

Improvements in immune function after mindfulness training have also been reported. In this regard, reduced psychophysiological stress and inflammatory responses (levels of the pro-inflammatory cytokine IL-6, C reactive protein, or in the expression of pro-inflammatory genes related to NF-κB factor) have been reported after mindfulness training [35–37]. For instance, there is some evidence of beneficial effects of mindfulness training on cell-mediated immunity in patients with cancer [38], and HIV [35, 39]. Studies with larger samples and more adequate methodologies (e.g., using more rigorous control groups, longer follow-ups) are needed to better ascertain the effects of MBSR on inflammatory processes. A recent meta-analysis [40] examined the impact of mind-body treatments (including meditation, Tai Chi, Qi Gong, and Yoga) on inflammatory markers, finding that when all results from healthy and heterogeneous clinical samples were pooled, there was some evidence supporting an anti-inflammatory effect of mind-body treatments.

There is currently a total absence of empirical evidence regarding the cost-effectiveness of MBIs for FMS syndrome. Worldwide, mindfulness research is rapidly expanding and being applied in different contexts, and there has been a call for more health economics research. Bearing in mind that costs are of crucial importance for policy-makers, who usually consider as first-choice treatment those interventions with the lowest cost per quality-adjusted life-year (QALY), economic evaluations of MBIs in FMS should be carried out to ensure (if cost-effective) their implementation in healthcare systems. Moreover, the evidence for mindfulness as the causal factor in structural and functional brain changes [27, 32] and the mechanistic effects of inflammatory markers on the therapeutic effects of mindfulness remain tenuous, so further work is needed to gain a deeper understanding of how mindfulness actually works.

Taking these issues into consideration the aim of this 12-month randomised, controlled trial (RCT) is three-fold: firstly, to examine the effectiveness and cost-utility for FMS patients of MBSR as an add-on to treatment as usual (TAU) versus a psychoeducational programme (FibroQoL) that recently that has been shown to be a cost-effective adjuvant to TAU [41]. Secondly, to examine pre-post neurobiological changes in brain structure and function, as well as in levels of some pro- and anti-inflammatory markers in the three study arms (TAU vs TAU + MBSR vs TAU + FibroQoL). Thirdly, to analyse the role of specific process variables (facets of mindfulness, pain catastrophism, psychological inflexibility, and self-compassion) as mediators of long-term clinical improvement.

## Methods

### Study design

This RCT protocol was developed following the Standard Protocol Items: Recommendations for Interventional Trials (SPIRIT) [42] and was recorded in the ClinicalTrials.gov trial register in September 2015 (NCT02561416). We designed a 12-month, parallel group, randomised (using a computer-generated randomisation list), single-blind, controlled trial (RCT) with three treatment arms. The Consolidated Standards of Reporting Trials 2010 (CONSORT) [43] and the Consolidated Health Economic Evaluation Reporting Standards (CHEERS) [44] will be followed. The three treatment arms are:

1. TAU + MBSR
2. TAU + FibroQoL
3. TAU

Patients from the three study arms will receive TAU; given that MBSR and FibroQoL were originally developed as complements to usual care, not as substitutes for it. Fifty percent of the participants in each study arm will undergo neurobiological evaluations (neuroimaging and inflammatory markers).

### Participants

A total of 180 FMS patients will be recruited from the Rheumatology service at Sant Joan de Déu Hospital (St. Boi de Llobregat, Spain). Sample size is established on the basis of a previous meta-analysis of controlled MBSR trials [45] in which a mean effect size of *d* = 0.53 was found. This effect size results in 1-b = 0.89 (alpha = 0.05) for *N* = 60 patients per group (180 patients overall). With 20 % maximal attrition, the power remains 1-b = 0.82. The estimated sample size for the study of biomarkers (neuroimaging and cytokines) was 30 participants per condition, this sample size is considered adequate given that approximately 20–40 participants (in between-group designs) are necessary to detect a change of 15 % in cerebral blood flow in regions of interest (ROI) by means of pseudo-continuous Arterial Spin Labeling (pCASL) [46].

### Eligibility criteria and Multi-stage recruitment process

For this study, we use a database with the medical records of FMS patients referred from local general practices to the Rheumatology Service at Parc Sanitari Sant Joan de Déu to confirm diagnosis. The sample pool consists of all FMS patients included in this database between January 2010 and September 2015 (N_sample pool_ = 531). Thus, all patients in the database were diagnosed with FMS (according to the American College of Rheumatology, ACR 1990 criteria) by rheumatologists from Parc Sanitari Sant Joan de Déu.

Patients meeting the following criteria will be eligible: 1. Patients of both genders between 18–65 years-old. 2. Able to understand the Spanish language. 3. Provide informed consent to participate. The following *general exclusion criteria* will be applied: 1. Participation in other RCTs. 2. Presence of cognitive impairment according to the Mini Mental State Examination (MMSE < 27). 3. Receiving psychological treatment during the previous or current year. 4. Reporting previous experience in meditation or other mind-body therapies. 5. Comorbidity with severe mental or medical disorders which interfere with treatment (severe medical illness, psychotic symptoms, substance abuse). 5. Unable to attend group sessions. 6. Pregnancy. 7. Involved in ongoing litigation relating to FMS.

Usual contraindications for scanning at 3Tesla and for measuring cytokine levels have been taken into account to establish the following additional inclusion/exclusion criteria for the biomarkers sub-study:

- *Inclusion criteria*: 1. Female gender. 2. Right-handed.
- *Exclusion criteria*: 1. Infection/cold symptoms on the day of blood extraction. 2. Needle phobia. 3. BMI > 36 kg/m^2^ or weight > 110Kg. 4. Neoplastic illnesses, infection, cardiopulmonary, vascular, or other internal conditions (collected from the medical history). 5. Use of oral or local corticosteroids or anticytokine therapy. 6. Consuming more than 7 caffeine units per day (1 caffeine drink will be permitted on the day of the study. 8. Smoking more than 5 cigarettes per day (no smoking will be permitted on the scanning visit days prior to scanning or whilst in the centre). 9. Cannot be evaluated by means of magnetic resonance imaging (MRI) (due to claustrophobia, metal implants, pace-makers, etc.). 10. Acute pain not -related to FMS on the day of the study (e.g. headache, lumbar pain). 11. Being pregnant or breastfeeding. It is important to point out that patients presenting neuroradiological alterations during MRI at baseline will be excluded from the sub-study. Patients will be allowed to continue with their stable medical treatment although, to reduce the effects of medications on biomarkers, they will be required to refrain from taking occasional (rescue) analgesic drugs (e.g., non-steroidal anti-inflammatory drugs, paracetamol) 72 h prior to the MRI and blood extraction. Changes in pharmacological/non-pharmacological treatment will be monitored throughout the study and may be a cause of drop-out from the final analyses.

### Procedure

The potential FMS participants will be screened through an initial telephonic interview by one of the authors (A.F.S), who will provide a general overview of the study. Subsequently, two highly trained clinical psychologists (not involved with the treatment and blind to group allocation) will make an appointment with those patients who met the inclusion criteria and agreed to participate in the study. The psychologists will check all inclusion/exclusion criteria (both general and additional for the biomarkers study) and will perform all face-to face interviews. As mentioned above, study participants will be randomised to one of the three conditions (i.e., TAU, TAU + MBSR, TAU + FibroQoL). Both MBSR and FibroQoL are conducted in a group format (up to 15 people in each group). To balance the number of participants eligible for the biomarkers sub-study across the three arms, stratified randomisation will be performed. The proportion of eligible participants for the biomarkers evaluation and those only meeting criteria for the general study will be pre-fixed at 1:1 in all samples. Four treatment waves (*N* = 45 each) will be carried out to achieve the required final sample (*N* = 180).

**Fig. 1:**
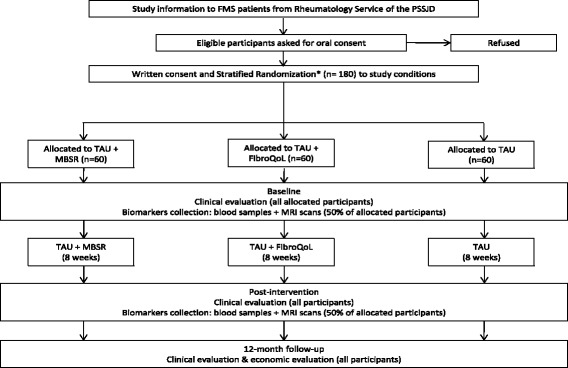
Flowchart of participants in the EUDAIMON study

Three-five days after the clinical evaluation, patients assigned to the biomarkers sub-study will be contacted for blood extraction and MRI completion. To minimise circadian variability in immunological markers, all whole blood samples will be collected between 8–9 am. Interventions will be conducted using a parallel design to reduce seasonal variability in the study measures. The participants will be interviewed at baseline, after treatment, and at 12-month follow-up (56 weeks after randomisation). The neuroimaging tests and cytokine measurements will be performed at baseline and after treatment. A flowchart of participants is displayed in Fig. 1.

### Interventions

#### *Intervention group* (TAU + MBSR; see Table 1)

**Table 1:** Session outlines for Mindfulness-Based Stress Reduction (MBSR) group treatment protocol

Session	MBSR^a^
1	Recognising the present moment. Beginner’s Mind, Introduction to Programme, Foundations of Mindfulness, More right with you than wrong, Introduction to Body Scan meditation, Intake.
2	Engaging with the breath. Patience, Basic Training, Working with perceptions, The Wandering Mind, Mindful Yoga.
3	Practice, practice, practice. Non-Striving, Mindfulness of Breathing Meditation, Lying Yoga, Attention vs. Disattention, Pleasant Events.
4	Stress and the flow of emotions. Stressful Events, Responding vs. Reacting, Seeing Our Patterns, Sitting Meditation, Standing Yoga, Research on Stress & Stress Hardiness.
5	Stress and thoughts: finding another place to stand. Working with Thoughts & Emotions, Group Reflections on Halfway Point, Small & Large Groups, Sitting Meditation, and Qigong.
6	Interpersonal mindfulness/mindful communication. Mindfulness & Communication, Avoiding Difficulty vs. Entering and Blending, Loving kindness Meditation, Walking Meditation. Daylong Session^a^: Putting it All Together.
7	Applying mindfulness. Sitting Meditation, Qigong, Trust & Self-Reliance, Mindful Consumption, Making the Practice Your Own, Mindfulness in Everyday Life.
8	Making mindfulness a part of your life. Sitting Meditation, Walking, The Class Never Ends: Practice for the rest of Your Life, Course Evaluations, Group Reflection & Checking Out.

^a^NOTE: Daily home practice of 30–45 min. duration, 6 days per week is encouraged (home practice guided by a workbook and audiotapes). In accordance with the MBSR protocol developed at the University of Massachusetts Medical School, 1 day-long meditation retreat will be held between sessions 6 and 8 of the programme

We will use the MBSR protocol developed at the University of Massachusetts Medical School, USA. MBSR [47] is intensive, structured training in mindfulness meditation which aims to help patients increase their awareness of the present experience and to relate to their physical and psychological conditions in a more accepting and non-judgmental way. The standard MBSR programme consists of 8 weekly sessions of around 2.5 h each and homework for 45 min a day, 6 days a week, even though several modifications in sessions, homework, and total duration can be observed among distinct courses for various patient populations. MBSR usually has three main components including a “*body scan*”, which involves a gradual sweeping of attention through the entire body from feet to head, focusing non-critically on any sensation or feeling in body regions and using periodic suggestions of breathing awareness and relaxation; “*sitting meditation*” which involves both mindful attention to breathing or on the rising and falling abdomen as well as other perceptions, and a state of non-judgmental awareness of cognitions and of the stream of thoughts and distractions that continuously flow through the mind; and “*Hatha yoga*” practice, which includes breathing exercises, simple stretches, and postures designed to strengthen and relax the musculoskeletal system. In addition to in-class mindfulness exercises, participants are encouraged to engage in home mindfulness practice and attend an all-day intensive mindfulness meditation retreat. The main premise of MBSR is that with repeated training in mindfulness meditation, individuals will eventually learn to be less reactive and judgmental toward their experiences, and more able to recognise, and break free from, habitual and maladaptive patterns of thinking and behaviour. It is important to mention that MBSR is not directed at symptom reduction but more fundamentally towards altering how perceptible mental processes and content are experienced, towards greater awareness, acceptance, and tolerance of the unavoidable vagaries of life, which facilitate enhanced psychological wellbeing, even in the face of continued symptoms. The eight 2 h MBSR sessions (once a week) will be performed in groups (*n* = 15 per group) and will be led by four accredited MBSR instructors (one per group) that have undergone MBSR training, which will enable an analysis of possible instructor effect on the outcomes, as recommended by Öst [48]. A book with MBSR manual and CDs with guided mindfulness practice sessions will be provided to the participants. In addition, we will reinforce MBSR treatment adherence by inviting participants to use a Mindfulness smartphone app developed in 2014 at the University of Zaragoza, Spain. The app has been scientifically validated by the Aragon Institute of Health Sciences. It is the first app that tracks the state of mindfulness and provides feedback on how mindful the person is during mindfulness meditation sessions. It also provides instruction in several mindfulness techniques by means of well-designed videos and audio recordings.

#### *Treatment as Usual control group* (TAU)

In Spain, treatment provided is mainly pharmacological and adjusted to the symptomatic profile of the patients with FMS. Counselling about aerobic exercise adjusted to patients’ physical limitations is usually provided.

#### *Active control group* (TAU + FibroQoL)

The FibroQoL is a psycho-educational programme for FMS patients based on a consensus document drawn up by the Health Department of Catalonia and shown to be cost-effective in a previous study [41]. This group-based programme is divided into two parts: The first part is led by three general practitioners, one rheumatologist and one psychologist. It includes updated information about pathophysiology, diagnosis, and management of FMS symptoms (4 sessions). The second part is led by a psychologist and includes training in self-hypnosis (4 sessions). In each of these sessions, a distinct self-hypnosis technique is explained and practiced. The goal of these techniques is to generate a state of deep relaxation, explore the sensations produced by one’s own body, achieve control over the body and pain, and imagine the one’s life in the future without pain. Many authors have emphasised the need to consider aspects of identity in order to understand the phenomena of chronic pain [49]. Therefore, it is especially useful to explore all the connections between the identity of a person, the perception of his/her body, and attitudes towards therapy in general. If we focus on the theory that each human activity is a process of creating meaning [50], people should assemble informal theories about, for instance, themselves, people or health. Their reactions to events such as bodily sensations or interpersonal experiences are mediated by their interpretations [51].

Some clinicians and scholars [51] highlight certain personality traits in patients with FMS, including an excessive sense of responsibility, extreme effort to please others, personal sacrifices made for others, little time set aside for themselves, and difficulty setting limits for others. These characteristics, as well as other difficulties showed by these patients, are discussed within the group and are part of the hypnotic inductions for each session. The group context has been considered an implementer of hypnotic response since the 18th century; the fact that someone in the group evidences minimal response raises expectations among the others and this reaction can increase the hypnotic susceptibility of all group members. The *naturalistic hypnosis* method requires that therapist is well trained in observation and should have a high degree of sensitivity when using the material provided by the patients. The reference model is Ericksonian hypnosis [52]. According to Erickson, a patient can use his/her own life experience, resources and prior learning therapeutically. The focus of Ericksonian hypnosis is to breakdown non-useful patterns and/or limit behaviours that prevent the construction of new meaning. Some typical properties of hypnosis facilitate this work, for example:

- Experience attention absorption: the person is absorbed in an aspect of context, stops paying attention to irrelevant stimuli.
- Changing the intensity of the experience: the sensorial and emotional experiences, linked to memories, are stronger.
- Willingness to experience: people during hypnosis, people are more amenable to unintentionally experience new perspectives; this feature is defined as suggestibility.
- Flexibility of Time-Space Relationship: experience of distortion of subjective time or temporary phenomena such as regression or projection into the future.
- Alteration of sensory experience: the person may experience changes in visual perception (e.g. *tunnel vision*), in hearing, in physical sensations (anesthesia, heaviness, lightness, size and position of body parts), loss of movement, or hands and/or fingers may occasionally move involuntarily (Table 2).

**Table 2:** Session outlines for the FibroQoL group treatment protocol

Session	FibroQoL^a^
1	Introduction and general information. Patients’ Expectations. History and epidemiology of the syndrome. Common symptoms in FM. Physiological mechanisms involved in the genesis of pain.
2	Collect information on the goals of each patient, explain differences between physical and emotional pain, clarify differences between hypnosis and self-hypnosis, administer hypnotisability test, hypnosis “safe place”.
3	Diagnosis and prognosis. Pharmacological and non-pharmacological treatments. Current model of health care in Catalonia and units specialised in the treatment of FM patients.
4	Discussion of goals and the difficulties that obstruct them, emphasize common personality characteristics, highlight exceptions to the problem, hypnosis “candle and bubbles”.
5	Strategies to increase self-esteem and regulate emotions. Pain experience and recurrent invalidation. Social support from family and close friends.
6	Exploration of possible changes, difference between acute and chronic pain, hypnosis: “imagination of a journey”.
7	Reviews the goals, ask for a future possible change (the miracle question), commitment to consolidation of the changes, hypnosis: “watch a photo album”.
8	Benefits of physical exercise in FM and closing remarks

^a^NOTE: The current FibroQoL version contains eight 2-h sessions instead of 9. The multidisciplinary team decided to dismantle the FibroQoL program, eliminating the session focused on “Holistic Medicine” because it contained some information about meditation/mindfulness. Thus, both MBSR and FibroQoL are structurally equivalent, which provides a comparison of MBSR with an intervention that matches MBSR in non-specific factors, but does not contain mindfulness ingredients

### Study measures

Study participants will complete the following instruments as part of a paper-and-pencil battery of measures (see Table 3):

- *Sociodemographic-clinical questionnaire*. The following information will be collected: gender, age, ethnic group, marital status, living arrangements, educational level, employment status, and annual income. Relevant clinical variables, such as family and personal medical history, years of FMS diagnosis and comorbid conditions will also be assessed.

**Table 3:** Study periods at which measures and data are collected

Study measures	Baseline	During treatment	2 months	12-months
Demographic information (gender, age, etc.)	X			
History of FMS (years of illness)	X			
Contact details	X			
SCID-I (depression module)	X			
*Screening measures*				
MMSE (cognitive impairment)	X			
*Primary outcome measure*				
FIQR (functional impairment)	X		X	X
*Secondary outcome measures*				
FSDC (fibromyalginess)	X		X	X
HADS (anxiety and depression)	X		X	X
PSS-10 (perceived stress)	X		X	X
MISCI (subjective cognitive impairment)	X		X	X
EQ-5D-5L (quality of life)	X			X
CSRI (health economic evaluation)	X			X
FFMQ^a^ (mindfulness facets)	X		X	X
PCS^a^ (pain catastrophizing)	X		X	X
PIPS^a^ (psychological inflexibility)	X		X	X
SCS-12^a^ (self-compassion)	X		X	X
*Other measures*				
Structural + Functional Neuroimaging data	X		X	
Inflammatory data	X		X	
CEQ (Expectation/Satisfaction with treatment)	X (EXP)		X (SAT)	
PGIC & PSIC (Impression of change)			X	X
Treatment attendance form: Attendance at each scheduled MBSR/FibroQoL session		X		
Log of MBSR and FibroQoL practices		X		
Treatment fidelity (review of video-taped MBSR sessions) & monitoring of adverse events		X	X	X

*CEQ* Credibility/Expectancy Questionnaire, *CSRI* Client Service Receipt Inventory, *EQ-5D-5L* EuroQoL questionnaire, *FFMQ* Five Facet Mindfulness Questionnaire, *FIQR* Revised Fibromyalgia Impact Questionnaire, *FSDC* Fibromyalgia Survey Diagnostic Criteria, *HADS* Hospital Anxiety and Depression Scale, *MISCI* Multidimensional Inventory of Subjective Cognitive Impairment, *MMSE* Mini-Mental State Examination, *PCS* Pain Catastrophizing Scale, *PGIC & PSIC* Patient Global and Specific Impression of Change, respectively, *PIPS* Psychological Inflexibility in Pain Scale, *PSS-10* Perceived Stress Scale, *SCID-I* Structured Clinical Interview for DSM Axis I Disorders, *SCS-12* Self-Compassion Scale

^a^Secondary outcome measures that also considered process variables

Inflammatory data = Cytokines Th1: IL-6, IL-8; cytokines Th2: IL-10; + high-sensitivity CRP test

Structural and Functional Neuroimaging = Voxel-Based Morphometry, Diffusion Tensor Imaging, pCASL, and rs-fMRI

#### Clinical characteristics and screening measures

- The *Mini-Mental State Examination* (MMSE) [53, 54] is a 30-item questionnaire designed to measure cognitive impairment that is widely used in older adults. The MMSE includes tests for orientation, memory, concentration, and visuospatial ability. In non-geriatric populations (≤65 years), such as our study sample, the threshold that suggests a “probable case” of cognitive impairment when the score is less than 27 points.
- The *Structured Clinical Interview for DSM Axis I Disorders* (SCID-I) [55]. Clinical diagnosis of mood disorders according to DSM-IV diagnostic criteria will be confirmed using the research version of the SCID-I (mood disorders module).

#### Primary outcome measure

- The *Revised Fibromyalgia Impact Questionnaire* (FIQR) [56, 57] includes 21 individual items that are all answered on an 11-point numeric rating scale from 0 to 10, with 10 reflecting greater impairment. The time frame is the previous 7 days and the items are distributed into three associated domains: “physical function” (9 items); “overall impact” (2 items that address the overall impact of FM on functioning and symptom severity); and “severity of symptoms” (10 items; pain, energy, stiffness, quality of sleep, depression, memory problems, anxiety, tenderness to touch, balance problems, and sensitivity to loud noises, bright lights, odours, and cold temperatures). The scoring system is straightforward: the physical function domain (0 to 90) is divided by 3, the overall impact domain (0 to 20) is not transformed, and the severity of symptoms domain (0 to 100) is divided by 2. The FIQR total score (0 to 100) is obtained by adding the three domain scores. The FIQR has been shown to be a psychometrically sound instrument, is clinically useful as it can be completed by patients in less than 2 min and scored in approximately 1 min, is recommended as a primary efficacy endpoint measure in FMS clinical trials, and is the “gold standard” assessment measure for multidimensional functional status in FMS patients [58].

#### Secondary outcome measures and process measures

- The *Fibromyalgia Survey Diagnostic Criteria* (FSDC) [59, 60] is a 6-item self-report questionnaire that registers the key symptoms of FMS according to the latest revision of the ACR. It includes assessment through the Widespread Pain Index (WPI) identifying 19 body areas (jaws, shoulders, upper arms, lower arms, hips, upper legs, lower legs, neck, chest, upper back, lower back and abdomen) where pain or tenderness was felt during the previous 7 days (total score 0–19). The Symptom Severity Scale (SS; range from 0–12) includes three major symptoms (fatigue, trouble thinking/remembering and waking up tired or unrefreshed), which is scored from 0 to 3, as well as three additional symptoms (pain or cramps in lower abdomen, depression, headache), which can be coded as present (1) or absent (0). The fibromyalgianess scale is defined as the sum of the WPI items (0–19) and the 6-item SS scale (0–12), so total scores can range from 0 to 31.
- The *Client Service Receipt Inventory* (CSRI) [61, 62] used for the present project will be designed to collect retrospective data on medication and service receipt: 1. Medication. A profile of the patient’s use of all prescribed medications (analgesics, non-steroidal anti-inflammatories, short- and long-acting opioids, etc.) will be requested, including the name of the drug, the prescriber, the dosage level, the total number of days taking the drug, the total dosage consumed, the reasons for changing the drug, and adherence. 2. Service receipt. We will collect information about emergency services, general medical in-patient hospital admissions and out-patient health care services. Patients will also be asked about type and number of diagnostic tests administered. As shown in Table 3, the CSRI will be administered at baseline and at a 12-month follow-up*; on both occasions, the previous 12 months will be reviewed*.
- The *EuroQoL-5D questionnaire* (EQ-5D-5L) [63] is a widely used health-related quality of life instrument with a non-disease-specific classification system composed of 2 parts: Part 1 is a self-reported description of health problems classified into five dimensions. Patients mark one of five degrees of severity in each dimension. The time frame is the day of responding. Part 2 records the current subject’s health on a Visual Analogue Scale (VAS); a vertical 10 cm line on which the best and worst imaginable health states score 100 and 0, respectively.
- The *Hospital Anxiety and Depression Scale* (HADS) [64, 65] was originally developed to quantify the severity of anxiety and depressive symptoms in non-psychiatric hospital patients The HADS includes a total of 14 items that assess anxiety (HADS-A) and depressive (HADS-D) symptoms, with 7 items in each subscale. Each item is answered on a four-point (0-to-3) scale so that possible scores range from 0 to 21 for both anxiety and depressive symptoms, with higher scores indicating greater severity.
- The *Perceived Stress Scale* (PSS-10) [66] is a 10-item, self-administered instrument that measures the degree to which situations in one’s life are considered stressful. Scores range from 0 to 72. The Spanish version of the PSS-10 has adequate reliability and validity [67].
- The *Multidimensional Inventory of Subjective Cognitive Impairment* (MISCI) [68] is a 10-item measure of perception of cognitive dysfunction in FMS, developed through classical test theory and item response theory from cognitive functioning item banks that were developed as part of the Patient Reported Outcomes Measurement Information System (PROMIS). The MISCI showed excellent internal reliability, low ceiling/floor effects, and good convergent validity with a similar measure.
- The *Pain Catastrophising Scale* (PCS) [69, 70] is a 13-item questionnaire derived partially from the Coping Strategies Questionnaire and other descriptions of catastrophising. It has three dimensions: Rumination (tendency to focus excessively on pain sensations), Magnification (tendency to magnify the threat value of pain sensations) and Helplessness (tendency to perceive oneself as unable to control the intensity of pain). The PCS total score and subscale scores are computed as the algebraic sum of ratings made for each item. PCS items are rated in relation to frequency of occurrence on 5-point scales (0 = never ~ 4 = almost always).
- The *Five Facet Mindfulness Questionnaire* (FFMQ) [71, 72] consists of 39 items that assess five facets of mindfulness. Items are rated on a Likert scale ranging from 1 (never or very rarely true) to 5 (very often or always true). The factors include: *Observing*, including noticing or attending to internal and external experiences such as sensations, thoughts, or emotions. *Describing* refers to labelling internal experiences with words. *Acting with awareness* involves focusing on one’s activities in the here and now as opposed to behaving mechanically. *Non-judging of inner experience* refers to taking a non-evaluative stance toward thoughts and feelings. Finally, *non-reactivity to inner experience* is allowing thoughts and feelings to come and go, without getting caught up in or carried away by them. In a recent online survey answered by 4,986 FMS patients from all 50 US states and 30 countries, the FFMQ subscales was shown to discriminate well among FMS patients self-identified as meditators or non-meditators. Overall, FMS patients with higher mindfulness scores had lower levels of FMS symptom severity as measured with the FIQR [73].
- The *Self-Compassion Scale-short form* (SCS-12) [74, 75] is a shorter version (12 items) of the SCS (26 items). The SCS was designed to assess overall self-compassion (total score) and components of self-compassion across three dimensions: common humanity, mindfulness and self-kindness. The SCS–SF shows good internal consistency (Cronbach’s α ≥0.86) and very high convergence with the long form of the scale (r ≥0.97).
- The *Psychological inflexibility in pain scale* (PIPS) [76, 77] is a 12-item scale designed to measure psychological inflexibility in pain patients. The instrument includes two factors: *avoidance* and *cognitive fusion* related to pain. The items consist of different statements that are considered to be related to chronic pain, psychological inflexibility, suffering and disability (coherent with the ACT theory). All the items are rated on a 7-point Likert-type scale that ranges from “1 = never true” to “7 = always true”, with higher scores indicating more psychological inflexibility.

#### Other measures

- The *Patient Global Impression of Change (PGIC) and Pain Specific Impression of Change* (PSIC) [78] are measures that have been frequently used as indicators of meaningful overall change [on a 7-point Likert scale, from 1 = “Much better” to 7 = “Much worse”] in treatments for chronic pain, whereas the PSIC asks about the impression of change in more specific domains: physical and social functioning, work-related activities, mood, and pain.
- *Adapted version of the Credibility/Expectancy questionnaire* (CEQ) [79]. The CEQ is a 6-item questionnaire for measuring treatment expectancy and credibility for use in clinical outcome studies. The questionnaire shows overall high internal consistency (α = 0.84–0.85). We will use an adapted version that evaluates Expectancy (at the end of the first treatment session) and Opinion (at the end of the last treatment session) about MBSR and FibroQoL.
- *Log of out-session for MBSR and psychotherapeutic practices*. An *ad hoc* instrument for the weekly recording of formal and informal mindfulness or FibroQoL home practice. A recent systematic review of systematic reviews and a meta-analysis of standardised mindfulness interventions (MBSR and MBCT) indicated that an increase in total minutes of mindfulness practice and class attendance are associated with a reduction of stress and mood complaints [80].
- *Adverse events of the interventions. Ad hoc* measure to check for adverse events (e.g., anxiety, dizziness, sleep problems, etc.) across the interventions and follow-up.

##### Neuroimaging

MRI will be performed on a 3.0 T Phillips Ingenia wide-bore MR scanner (70 cm bore size) fitted with an 8-channel, phased-array receive-only head coil. T1- and T2-weighted images will be acquired for radiologic assessment and image registration. We want to explore voxel-wise structural and functional changes associated with participation in the different treatments across the brain and in functionally-defined brain regions of interest relating to meta-awareness, body awareness, memory consolidation-reconsolidation, and emotion regulation. The main ROI for structural and functional datasets are defined as primary and secondary somatosensory cortices, anterior cingulate cortex, thalamus, insula, amygdala, hippocampus, frontal pole and orbitofrontal cortex. These ROI will be delineated with the aid of a neuroimaging atlas such as the WFU Pickatlas [81]. Prior to and after MRI, all participants will be asked to rate perceived pain intensity using a horizontal visual analog scale (100 mm length) marked “no pain” and “maximum pain” at the endpoints. The time frame is “the day of responding” and “during the MRI session”, respectively.

##### Brain structure

Our intention is to use longitudinal *voxel-based morphometry* (VBM), an automated structural MRI analysis technique, to examine whether the three interventions (TAU, TAU + MBSR, TAU + FibroQoL) produce local changes in grey matter. We will acquire high-resolution T1-weighted 3-dimensional volume scans pre and post treatment to assess for the presence of longitudinal change following treatment. Specifically, relative local increases and decreases between pre- and post-treatment scans will be compared within and between study groups. In addition, we will use *Diffusion tensor imaging* (DTI). The diffusion MR data will be analysed by using the diffusion tensor model. After a mathematical diagonalisation process, the eigenvectors and eigenvalues describing the tensor ellipsoid will be determined. Then, two standard diffusion indices will be obtained: the apparent diffusion coefficient and the fractional anisotropy. DTI scans pre and post treatment will permit to assess changes in axial diffusivity, with lower values being interpreted as structural enhancement of white matter. For example, lower axial diffusivity at post-treatment in MBSR participants compared to control participants would suggest a white matter increase as consequence of meditation training.

##### Brain function

Recent evidence [82] has demonstrated the suitability of pseudo-continuous arterial spin labeling (pCASL), a perfusion MRI technique, to quantify changes in regional cerebral blood flow (rCBF) that relate to the experience of ongoing persistent pain. In this study we will assess rCBF prior to and following the three treatments. pCASL data acquisition, pre-processing, and analysis methodologies have been published [82, 83], as applied to various disease states and therapeutic regimens, using gold-standard software packages (SPM-8 and FSL). We will acquire pCASL images using a gradient echo single shot echoplanar imaging readout resulting in whole-brain blood-flow maps. We will analyse pre-post rCBF differences within and between groups, and whether pre-post rCBF differences are related to variability in subjective ongoing pain.

Multiple resting-state networks have been found which show activity during rest and during tasks. One of them is the DMN, which shows a decrease in activity during cognitive tasks and is activated during self-referential thinking. Napadow et al. [12] showed that resting connectivity between the insular cortex (region involved in pain perception) and the DMN is significantly correlated with clinical pain at the time of the scan in FMS patients. In this work, we will use resting-state fMRI to compare changes in insular cortex-DMN connectivity between study conditions as well as to analysing any correlation between this connectivity and clinical pain. During the 8-min resting state fMRI acquisition period, the FMS patients will be instructed to remain awake with their eyes closed.

##### Inflammatory markers

Blood samples will be collected and serum will be stored at −80° for biochemical analyses. Levels of the following inflammatory markers will be assessed: IL-6, IL-8, IL-10, and high-sensitivity C-reactive protein [84]. Milliplex® assay kits (Human High Sensitivity T Cell kit) from MerkMilliporte (commercial firm) will be used for cytokine quantification in a Luminex® platform. CRP-hs will be assessed by turbidimetry in an Olympus AU5400 Autoanalyser.

### Statistical analyses of study outcomes

SPSS v22.0, STATA v13.0 and Mplus v7.2 will be used for the statistical analyses. One-way ANOVAs (with post hoc Tukey’s HSD or Games-Howell tests) for continuous values and *χ*^2^ tests with continuity corrections for categorical values will be computed on all baseline measures and socio-demographic variables to examine pre-treatment differences between groups.

#### Analysis of clinical efficacy

To assess treatment effects, we will perform Intention-to-Treat analyses, which will include all participants who undergo random allocation, using multiple imputation to replace missing values.

To study the differences in the primary outcome and in each of the secondary outcomes between groups, linear mixed-effects models [85] will be prepared using Restricted Maximum Likelihood to estimate the parameters. Comparisons amongst treatment groups at post-treatment and at 12-month follow-up will be computed with estimates derived from the linear mixed-effect models (use of the Bonferroni method to adjust the significance level of the pairwise contrasts). Calculations of between-groups effect sizes using Cohen’s *d* with its 95 % confidence interval will be based on the pooled standard deviation at baseline. Finally, to make the findings from the present RCT meaningful to clinicians, the number-needed-to-treat (NNT) [86] in the MBSR condition will be reported. Taking the Luciano et al. [87] NNT approach as our reference, we dichotomise participants into responders or non-responders using three separate cut-off criteria: (a) ≥ 20 % reduction on the FIQR total score from baseline to post-treatment; (b) ≥ 50 % reduction on the FIQR total score from baseline to post-treatment; and (c) number of patients crossing a cut-off point (reaching no worse than mild functional impairment; FIQR total score < 39).

Given that some pharmacological treatments may interfere with cytokine levels [88], differences in cytokine levels between patients taking vs. not taking antidepressants will be evaluated.

#### Mediational analyses

We will examine whether the effect of treatments on outcomes at 12-month follow-up are mediated through changes in “*process variables*” (mindfulness, pain catastrophising, psychological inflexibility, and self-compassion) at post-treatment. Using two-stage meta-analytic structural equation modelling, Gu and colleagues [89] identified moderate and consistent evidence for some of the proposed process variables [mindfulness and rumination (dimension of catastrophising)] and preliminary but still insufficient evidence for others [self-compassion and psychological flexibility] as mediator mechanisms underlying standardised MBIs (MBSR and MBCT). Following Luciano et al. [87], pre- to post-treatment change scores for the process variable, and the pre- to follow-up change scores for the outcome variables will be computed. We will analyse the direct and indirect relationships between treatments (TAU + MBSR vs. TAU + FibroQoL), process variables, and study outcomes using path analysis models. The treatment condition is considered the independent variable, the pre-post change scores in the process variables are the mediators, and pre- to follow-up changes in the outcome variables are the dependent variables. In this way, we are taking temporality into account, which increases the prospect of establishing conclusions about causality. We will analyse the data of participants from the MBSR and FibroQoL treatments who receive a sufficient dose of the intervention, defined in this case as attendance at a minimum of 6 of the 8 weekly sessions. Simple and multiple mediation (simultaneously testing multiple variables as mediators) models will be computed. The direct path between study condition and clinical outcome and the indirect effect through the process variables will be tested.

#### Cost-utility analysis

Taking a previous study by our group as a reference [90], the cost-utility of TAU + MBSR compared to the other study arms will be evaluated from healthcare and societal perspectives.

Costs will be estimated from the healthcare and societal perspectives during the 12 months of follow-up. Direct health care costs are calculated by adding costs derived from medication consumption, medical tests, use of health-related services, and cost of staff to run the intervention. The cost of medication is calculated by determining the price per milligram during the study, according to the Vademecum International (Red Book), and including value-added tax. Total costs of medications are calculated by multiplying the price per milligram by the daily dose in milligrams and the number of days receiving such treatment. The unit costs of medical tests and health services will be obtained from the SOIKOS health database (http://www.oblikue.com/bddcostes/). Indirect costs are calculated by taking the number of days on sick leave and multiplying it by the minimum daily wage in Spain. Finally, total costs are calculated by adding direct and indirect costs. The utilities represent the rating of the patients’ quality of life on a scale from 0 (as bad as death) to 1 (perfect health). QALYs will be calculated using Spanish EQ-5D-5L tariffs.

First, we will use a micro-costing approach, which involves careful specification of training costs, staffing costs, venue overheads, materials, and staff travel. Then, following the International Society for Pharmacoeconomics and Outcomes Research (ISPOR) core recommendations for cost-effectiveness analyses alongside RCTs [91], we will calculate the incremental cost-utility ratios, defined as the difference in mean costs divided by the difference in mean QALYs. As the duration of the study is 12 months, neither costs nor outcomes are subject to discounting. QALYs gained in each evaluation are approximated by using the area under-the-curve technique. To gain insight into the uncertainty around the pooled mean ICUR, we will plot the pooled bootstrapped cost-effect pairs on cost-utility planes. Finally, acceptability curves will be presented which represent the probability that the intervention is cost-effective, given a varying threshold for the willingness to pay for each QALY gained. The robustness of the cost-utility results will also be tested by computing distinct sensitivity analyses. For instance, we will perform a per protocol analysis from which the FMS patients who do not attend at least 6 MBSR or FibroQoL sessions will be excluded.

#### Neuroimaging analyses

Treatment-related differences in both structural and functional MRI datasets will be assessed using mass-univariate voxelwise General Linear Models. Thus, repeated measures ANOVA functions available in SPSS will be computed to examine between- and within-group effects of treatments. A standard threshold of *p* < 0.05 will be applied for ROI data, correcting for multiple comparisons.

### Ethical issues

Written informed consent will be obtained from all participants before randomisation. Participants will be provided with a general overview of the aims and characteristics of the study and the interventions before signing the informed consent. They will also be assured that they will be participating voluntarily and can withdraw at any time from the study with the guarantee that they will continue to receive the most appropriate medical treatment prescribed by their general practitioner. The study will be performed in accordance with the ethical standards laid down in the 1964 Declaration of Helsinki and its subsequent updates. The FSJD Research Committee Board evaluated and approved the study protocol in May 2015 (PIC-102-15). All patient data will be treated as confidential and only the research team will be allowed to access it after recodification of name and personal identity number (so no individual can be directly identified). Only the principal investigator of the study will have access to the code key which will be stored separately in a safe place in accordance with Spanish legislation. All data will be computer processed and stored. Blood samples will be stored, encrypted, and will not be directly traceable back to individuals. Blood samples will be used only in ways to which the participants consented and may only be made available to a new research project after Ethical Research Committee approval and participants provide a new agreement. Furthermore, the participants have the right to request, without explanation, that their samples be destroyed or made completely anonymous.

### Forecast execution dates

Initial recruitment of patients: January 2016

Finalisation of patient recruitment: January 2017

Finalisation of patient monitoring period: April 2018

Publication of results: December 2018

## Discussion

The study described here will evaluate, for the first time, the cost-utility of MBSR for FMS patients compared with usual care and with an empirically-validated intervention (FibroQol) which proved to be cost-effective in such patients in a high-quality RCT [41]. Therefore, this is a 3-armed RCT in which we include an active control condition (psycho-education) that permits us to control the “frustrebo response” (disappointment among control participants at not receiving treatment) associated with waiting-list and TAU conditions [92]. The RCT was designed following the CONSORT recommendations, the economic evaluation was planned following the updated ISPOR core recommendations for cost-effectiveness analyses alongside RCTs, the sample size in each study arm is sufficiently large and the follow-up period is sufficiently long to capture important costs and outcomes. If our trial results are sufficiently robust, MBSR programmes might be delivered as part of a strategic, coherent or appropriately resourced approach for patients with FMS in the healthcare context [80, 93]. According to Gotlink et al. [80], “*further research should also look more into the mechanisms whereby these therapies (MBSR-MBCT) are efficacious*”. In the present RCT, we are not only interested in the clinical effects of MBSR at long-term, but also in the “process” psychological variables and neurobiological changes that contribute to them, which is a current source of interest and debate in the scientific literature [89, 94].

One of the main risks in this study may be dropouts. A sensitivity analysis using a per protocol strategy will be performed to determine the impact of the adherence to the protocol in both the MBSR and FibroQoL groups. This RCT is intended to be as naturalistic as possible, so prescribed drugs are permitted throughout the study. The effects of these drugs on clinical and biological outcomes may constitute another study limitation. However, no baseline differences among study arms regarding medical drugs are expected (due to randomisation) and sub-analyses evaluating the potential effects of the main pharmacological treatments (e.g., antidepressants) on the outcomes will be performed. Moreover, received pharmacological treatment may not be stable over time (e.g., initiating antidepressant treatment due to a depressive episode, changing principle/dose of other drugs due to variations in clinical symptoms, or starting individual therapy with a psychologist) so it is probable that some patients will be excluded from the study due to drastic changes in their treatment. Patients will be asked at post- and follow-up assessments for details about changes in medical treatment received and the clinicians will check the pharmacological treatment history in the patients’ clinical chart.

To our knowledge, this study represents the first attempt to detect structural and functional brain changes and inflammatory marker variations after MBSR in patients with FMS. Indeed, few studies have focused on exploration of the neural and inflammatory underpinnings of psychological treatments in chronic pain patients. We hope that our work will offer new advances in understanding the psychophysiological processes altered by mindfulness and their relationship with clinical outcomes. Understanding of how mindfulness changes these variables will in turn increase our understanding of the role of the brain and the immune system in FMS. Furthermore, determining FMS profiles according to psychological and brain/immune variables may also allow prediction of treatment response in patients with this diagnosis. Given that patients with FMS usually present comorbidities with psychiatric disorders (such as depression or anxiety) and other central sensitivity syndromes (such as chronic fatigue syndrome), the results from this study may have translational value.

### Consent for publication

Not applicable.

### Availability of data and material

Not applicable.

## Abbreviations

ACR: American College of Rheumatology
CEQ: Credibility/Expectancy Questionnaire
CHEERS: Consolidated Health Economic Evaluation Reporting Standards
CNS: Central Nervous System
CONSORT: Consolidated Standards of Reporting Trials
CSRI: Client Service Receipt Inventory
DMN: Default Mode Network
DTI: Diffusion Tensor Imaging
EAN: Executive Attention Network
EQ-5D-5L: EuroQoL questionnaire
FFMQ: Five Facet Mindfulness Questionnaire
FibroQoL: Psychoeducational programme for improving quality of life in fibromyalgia
FIQR: Revised Fibromyalgia Impact Questionnaire
fMRI: functional Magnetic Resonance Imaging
FMS: Fibromyalgia syndrome
FSDC: Fibromyalgia Survey Diagnostic Criteria
HADS: Hospital Anxiety and Depression Scale
High-sensitivity CRP test: high sensitivity C-reactive protein
ICUR: Incremental Cost Utility Ratio
ISPOR: International Society for Pharmacoeconomics and Outcomes Research
MBCT: Mindfulness-Based Cognitive Therapy
MBIs: Mindfulness-based interventions
MBSR: Mindfulness-Based Stress Reduction
MISCI: Multidimensional Inventory of Subjective Cognitive Impairment
MMSE: Mini-Mental State Examination
MRI: Magnetic Resonance Imaging
NNT: number-needed-to-treat
pCASL: pseudo-continuous Arterial Spin Labeling
PCS: Pain Catastrophising Scale
PGIC: Patient Global Impression of Change
PIPS: Psychological Inflexibility in Pain Scale
PSIC: Patient Specific Impression of Change
PSS-10: Perceived Stress Scale
QALY: Quality-Adjusted Life-Year
rCBF: regional Cerebral Blood Flow
RCT: Randomised Controlled Trial
ROI: Regions of Interest
rs-fMRI: resting state functional Magnetic Resonance Imaging
SCID-I: Structured Clinical Interview for DSM Axis I Disorders
SCS-12: Self-Compassion Scale
SPIRIT: Standard Protocol Items: Recommendations for Interventional Trials
TAU: Treatment as Usual
VBM: voxel-based morphometry
